# Effect of ball collision direction on a wet mechanochemical reaction

**DOI:** 10.1038/s41598-020-80342-w

**Published:** 2021-01-08

**Authors:** Takahiro Kozawa, Kayo Fukuyama, Kizuku Kushimoto, Shingo Ishihara, Junya Kano, Akira Kondo, Makio Naito

**Affiliations:** 1 Joining and Welding Research Institute, Osaka University, 11-1 Mihogaoka, Ibaraki, Osaka 567-0047 Japan; 2 Institute of Multidisciplinary Research for Advanced Materials, Tohoku University, 2-1-1 Katahira, Aoba-ku, Sendai, Miyagi 980-8577 Japan

**Keywords:** Materials science, Techniques and instrumentation

## Abstract

Mechanochemical reactions can be induced in a solution by the collision of balls to produce high-temperature and high-pressure zones, with the reactions occurring through a dissolution–precipitation mechanism due to a change in solubility. However, only a fraction of the impact energy contributes to the mechanochemical reactions, while the rest is mainly consumed by the wear of balls and the heat generation. To clarify whether the normal or tangential component of collisions makes a larger contribution on the reaction, herein we studied the effect of collision direction on a wet mechanochemical reaction through combined analysis of the experimental reaction rates and simulated ball motion. Collisions of balls in the normal direction were found to contribute strongly to the wet mechanochemical reaction. These results could be used to improve the synthesis efficiency, predict the reaction, and lower the wear in the wet mechanochemical reactions.

## Introduction

Mechanochemical reactions of solid phases using grinding equipment are a facile, green, and scalable synthetic approach for organic^1,2^, inorganic^3^, metallic^4–6^, and energy fields^7,8^. When solid reactants are milled, they experience mechanical stress and strain from collisions and shears with the milling body, which induce solid–solid reactions including interfacial dislocations and ion diffusion^9–11^. Another driving force of these non-equilibrium reaction is local frictional heat at the solid surface. However, such heat could also decompose thermodynamically unstable hydrous materials including hydrates and hydroxides^12^. Besides, the control of particle shapes is very difficult while applying external forces. An alternative is wet mechanochemical reactions based on a dissolution–precipitation mechanism instead of solid–solid reactions^13–16^. By utilizing the solution-based reaction mechanism, we were able to control the particle shape in synthesized hydrates and hydroxides despite the use of planetary ball milling^17,18^. However, to improve the versatility of this method for synthesizing functional particles, it is necessary to determine how the input mechanical energy acts on the solution or reactants.

Chemical interaction in the mechanochemical process may be modelled by collision theory due to its statistical nature^19^. The reaction rate (*v*) in a mill can be estimated as

1 $$v = KxS$$

where *K* is a constant characterizing the reaction probability at a given mechanical action per unit contact, *x* is the probability for the particles to collide with a milling body per time, and *S* is the contact area of the different reactants during mechanical action. In solution, the dissolution and dispersion of reactants reduce the contact probability between them as compared to that with the milling body (such as ball media). Consequently, the rate of wet mechanochemical reaction mainly depends on *K*, which is the impact energy in the collision between balls. Such collision causes high-temperature and high-pressure local zones within the solution^20–22^. This limited reaction field affects the local solubility of reactants and products, which in turn can induce the dissolution, nucleation, and crystal growth of solid species. Mechanically, the collision of balls can be divided into normal and tangential components. The impact energy (*E*) is the sum of the two components:

2 $$E = e_{n} + e_{t}$$

where *e*_*n*_ and *e*_*t*_ are the impact energies in the normal and tangential directions, respectively. The grinding efficiency, defined as the energy to create a new surface for the energy input, is ~ 15% at best for the comminution of quartz and soda-lime glass^23^, while most of the input mechanical energy does not contribute to the reaction. Therefore, determining which collision direction contributes the most to a wet mechanochemical reaction can help estimate the reaction rate from the input mechanical energy, and predict the most efficient milling conditions for producing the desired particles. There has been no discussion of particle synthesis by wet mechanochemical reactions from the ball collision direction.

In this study, we examined how the ball collision direction affects a wet mechanochemical reaction by combining experimentally measured formation rates with simulated ball motion in solution. The model reaction was the formation of lithium titanate hydrate (Li_1.81_H_0.19_Ti_2_O_5_·*x*H_2_O, LHTO) through planetary ball milling of LiOH and TiO_2_ in water. The formation fraction of LHTO was measured as the treatment conditions were varied systematically. Meanwhile, our group had developed accessible simulation software (KIK DEM, ver.1.1) to reveal the motion of balls during wet milling, using the distinct element method (DEM) to treat the ball media and simplified modeling of interactions with fluids^24,25^. This software can output the collision frequency and the impact energies for each collision component. After fitting the formation fraction of LHTO and the calculated cumulative impact energy in each milling condition, the formation rate was found to follow the impact energy of the normal component, which increased linearly with the size and input of the balls. The values of the tangential component varied a great deal depending on the motion of balls in the milling vessel. However, the excess tangential impact energy was consumed without forming LHTO. The resulting guideline for wet mechanochemical reactions provides a highly efficient route for synthesizing thermodynamically unstable materials at room temperature.

## Results and discussion

**Figure 1:**
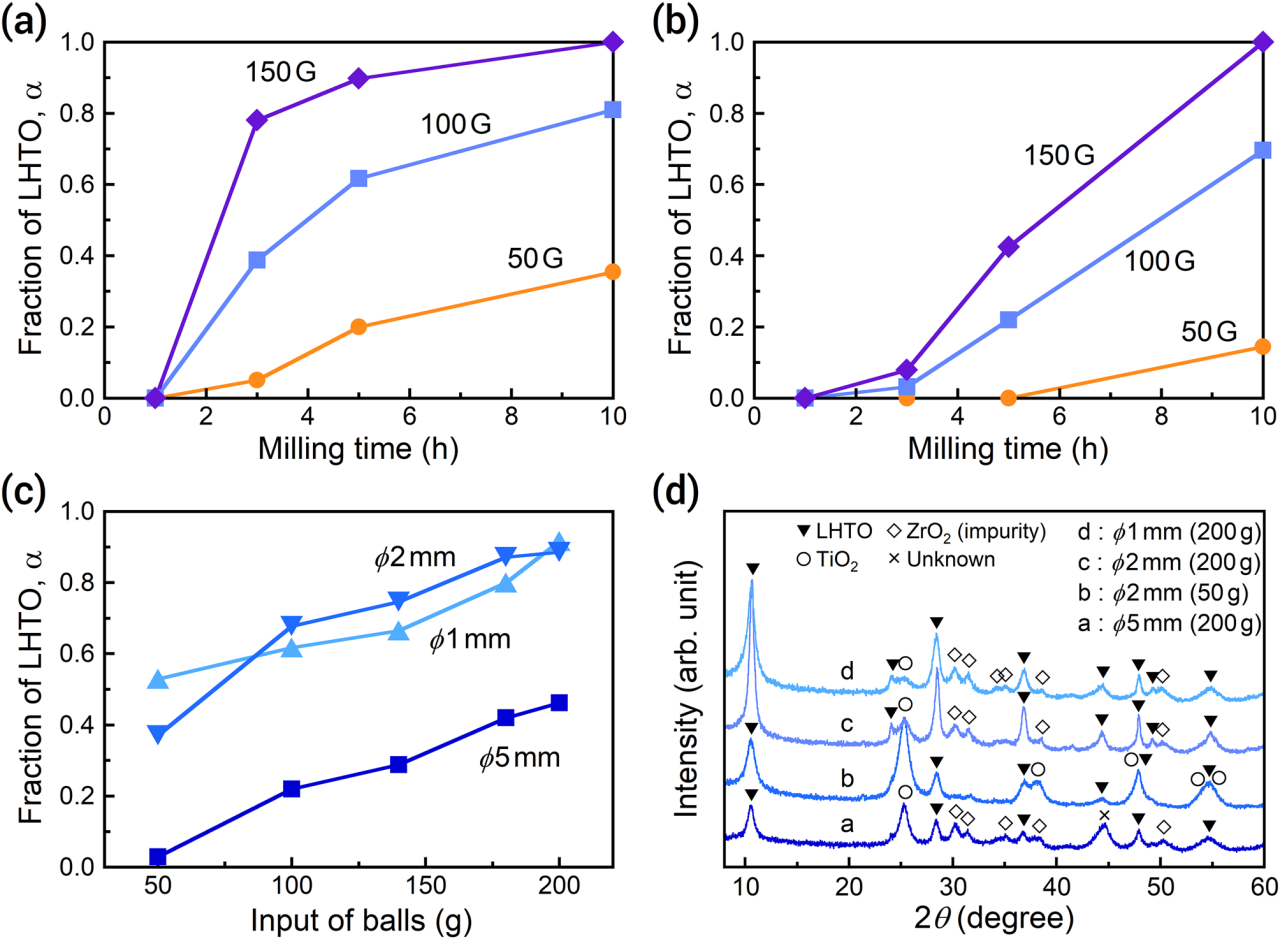
Formation of LHTO through the wet mechanochemical reaction. Formation fraction of LHTO after wet planetary ball milling at 50–150 G for 1–10 h with 100 g of balls at (**a**) *ϕ*1 mm and (**b**) *ϕ*5 mm. (**c**) Effect of ball input and size on the reaction at 100 G for 5 h. (**d**) XRD patterns of typical products obtained at 100 G for 5 h using balls of different sizes and inputs.

Many researchers have reported that LHTO is an attractive precursor of Li_4_Ti_5_O_12_ anode for Li-ion batteries, due to its superior properties for constructing various morphologies^26–28^. However, the conventional hydrothermal and solvothermal synthesis routes for LHTO suffer from certain technical drawbacks, including the need of highly reactive titanium sources, restricted temperature–pressure conditions, and prolonged treatment time for nucleation and growth^26–29^. In comparison, in the mechanochemical reaction, LHTO is formed from inexpensive TiO_2_ by using high-energy grinding at room temperature to induce its dissolution in the solution^17^. The normally insoluble TiO_2_ can gradually dissolve in a strongly basic LiOH solution^30,31^. The collisions of balls trigger the formation of LHTO in the solution, while the formation rate depends on the ball size. When relatively small balls (*ϕ*1 mm) are used, the formation fraction of LHTO (α, estimated by the X-ray diffraction (XRD) peak ratios of TiO_2_ and LHTO) increases rapidly with an increase in the centrifugal acceleration and milling time (Fig. 1a). The centrifugal acceleration (G) was calculated using technical data as revolution diameter and frequency of the motor and controlled by the revolution speed. The obtained result agrees with previous work using *ϕ*2 mm balls^17^. In contrast, the formation rate is lower for larger balls of *ϕ*5 mm (Fig. 1b). For example, under the milling condition of 150 G and 3 h, the value of α is ~ 0.8 for *ϕ*1 mm balls but only 0.1 for *ϕ*5 mm balls. When fixing the ball size at *ϕ*1 mm, α grows with time more slowly at low centrifugal accelerations. These results suggest that smaller balls improve the efficiency of LHTO formation.

The collision frequency per unit volume clearly depends on the ball size. Furthermore, an increase in ball input (i.e., more balls) also increases the collision frequency. The enhanced contact probabilities lead to more opportunity to form LHTO. Indeed, the α value increases with decreasing ball size and increasing ball input (Fig. 1c). As mentioned above, the formation rate is slow with larger balls when the other conditions are fixed. Under the milling condition of 200 g balls, 100 G, and 5 h, the α value reaches 0.9 for *ϕ*1 mm and *ϕ*2 mm balls but remains at 0.45 for *ϕ*5 mm balls. Although increasing the ball input accelerates the wet mechanochemical reaction, it also causes the generation of impurity due to wear. After milling with 50 g of *ϕ*2 mm balls, the product (α = 0.4) contained only LHTO and TiO_2_ (Fig. 1d). In contrast, a similar fraction of product could be formed using 200 g of *ϕ*5 mm balls, but the product also exhibited diffraction peaks of ZrO_2_ as an impurity. The generation of impurity is unavoidable even for small balls if their input is high.

Overall, the experimentally measured formation of LHTO through wet mechanochemical reaction revealed the following findings when the other reaction parameters are fixed in each case. (1) The product is formed faster when using smaller balls. (2) The formation fraction increases with an increase in the centrifugal acceleration and treatment time. (3) Increasing the input of balls promotes the reaction. (4) A larger amount of balls (regardless of their size) induces the generation of impurity due to wear. Based on these, we hypothesize that the collision frequency of balls during milling controls the formation rate of LHTO. Next, this hypothesis was examined using simulation, which assumed that only the balls and water were present in the ball mill.

**Figure 2:**
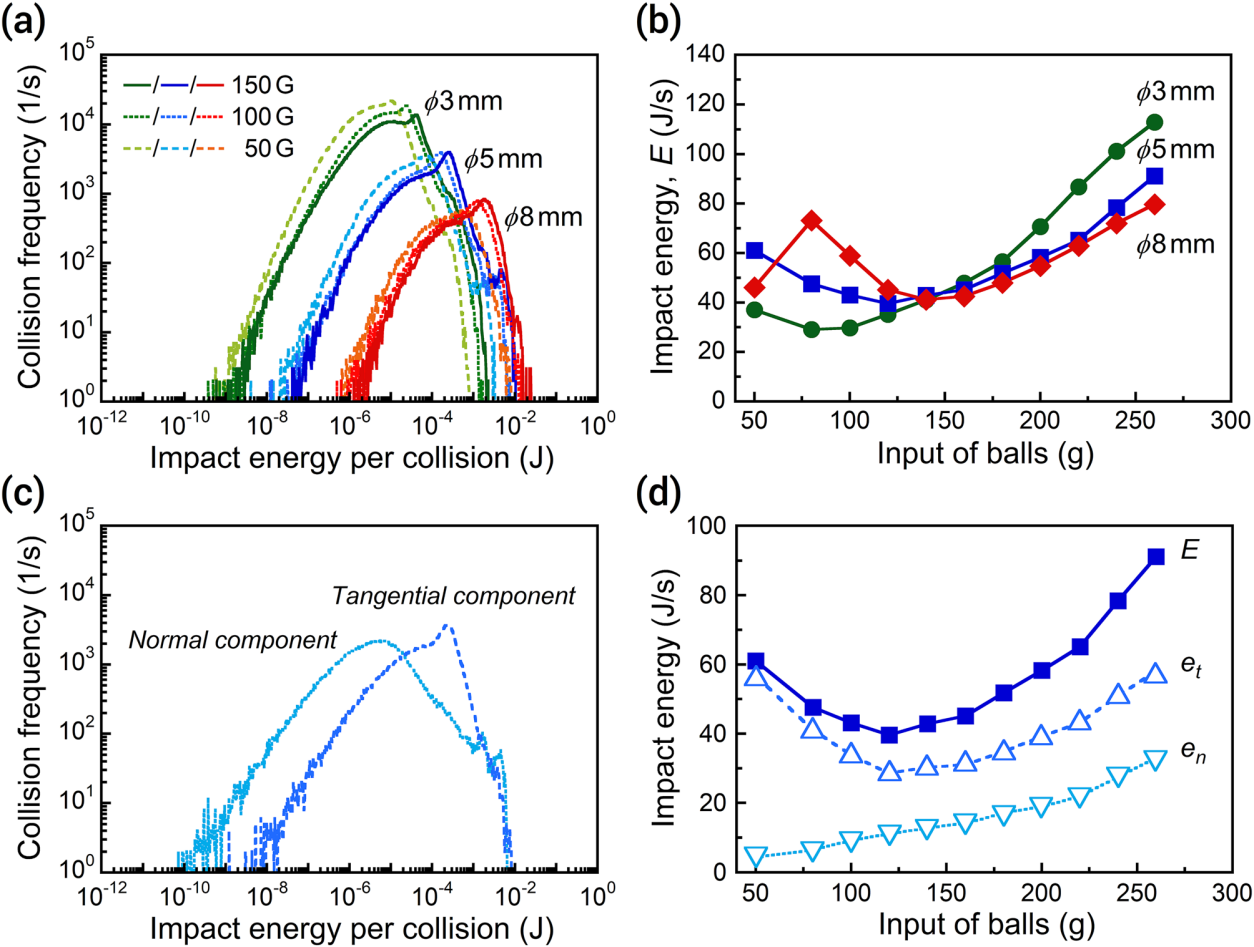
Impact energies between balls calculated for various planetary ball milling conditions in water. (**a**) Distributions of impact energy per collision at the ball input of 100 g. (**b**) Relationship between the input of balls (*ϕ*3–8 mm) and the total impact energy at 150 G. (**c**) Distributions of impact energies in the normal and tangential directions at 150 G with *ϕ*5 mm balls (100 g). (**d**) Relationship between the input of *ϕ*5 mm balls and the impact energies of each component at 150 G.

Firstly, we simulated the effects of centrifugal acceleration and ball size while fixing the ball input at 100 g (Fig. 2a). To reduce the computational load, the lower limit of ball size was set at 3 mm. With increasing ball size, the impact energy per collision increases whereas the collision frequency decreases. The distribution width of the impact energy is about three orders of magnitude larger for smaller balls. Increasing the centrifugal acceleration from 50 to 150 G leads to a shift of frequency distribution to higher regions, however the effect is less than that from changing the ball size. Next, we investigated the effect of ball input on the impact energy (Fig. 2b). The total impact energy (*E*) is an integrated value of the impact energy per collision and the frequency. *E* initially decreases with an increase in the ball input, and then it increases at above 100–140 g. However, the use of *ϕ*8 mm balls leads to a rapid rise of impact energy with the input up to 80 g, and then it decreases to a minimum value at 140 g. When an excess volume of balls is used, the impact energy is higher with smaller balls in Fig. 2b. The same trend was observed under lower centrifugal accelerations of 50 G and 100 G (Fig. S1).

**Figure 3:**
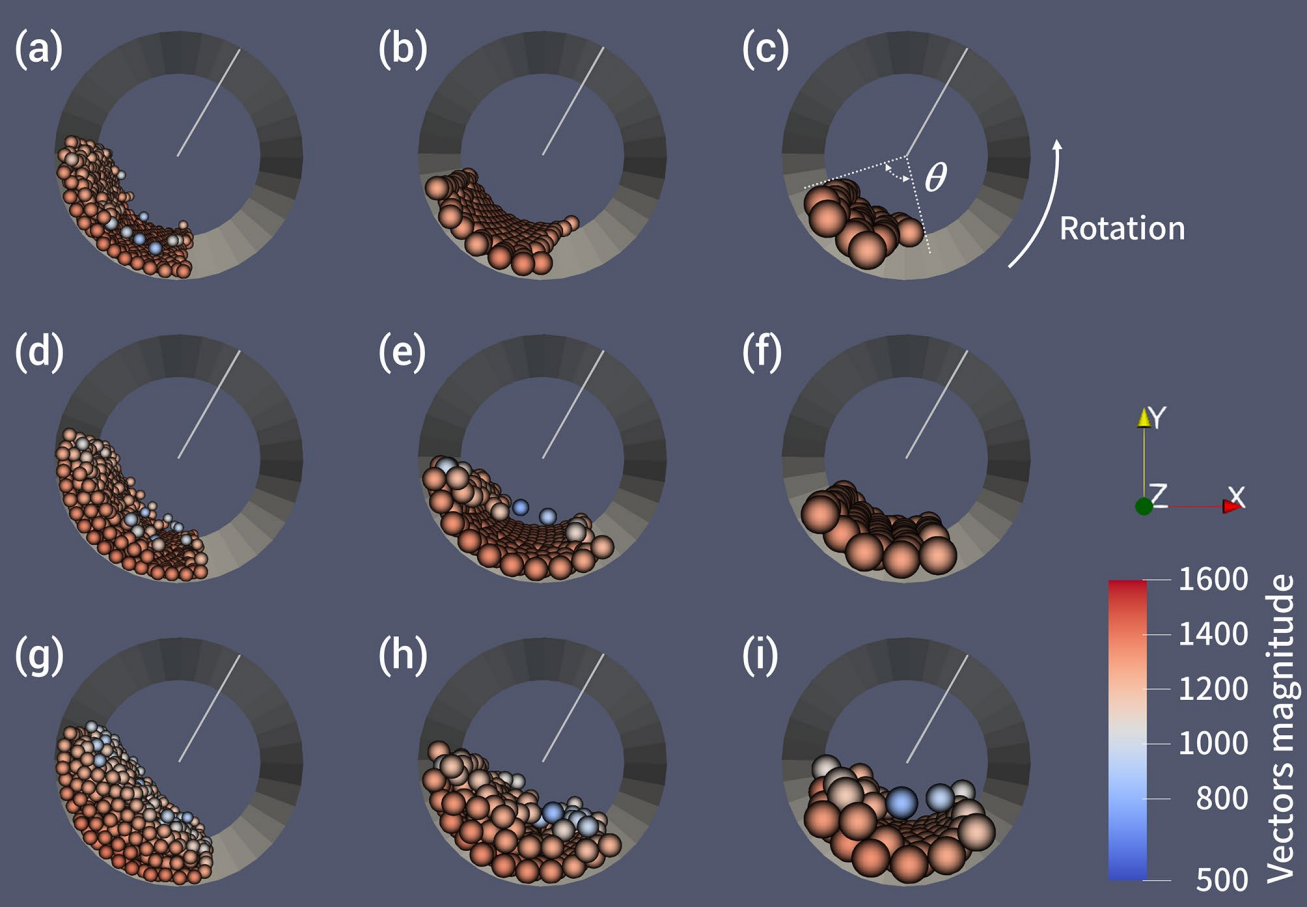
Snapshots of ball motion during the wet planetary ball milling at 150 G. Left column, *ϕ*3 mm; middle, *ϕ*5 mm; right, *ϕ*8 mm. Ball input: (**a**–**c**) 50 g, (**d**–**f**) 80 g, and (**g**–**i**) 140 g. The line from the center of the vessel indicates the direction of the center of revolution. Water is omitted from the visualization. Visualization of the simulated data was performed using ParaView software (ver. 5.8.0).

To clarify the dependency of impact energy on the ball size and the input, we visualized the ball motion during milling. Representative behaviors of the balls are displayed in the Movies (Supporting Information). The balls are pushed against the interior wall of the vessel due to the strong centrifugal force. Since the rotation and revolution directions in our planetary mill were the same, the circulation motion found in tumbling mill and inverse direction-type planetary mill^32,33^ did not occur in the simulation here. As a result, the collisions of balls are limited to a relatively small region in the packing layers pushed against the wall. The top-view snapshots of the interior of the vessel reveal the different packing states depending on the ball size (Fig. 3). The loading of 50 g *ϕ*3 mm balls leads to the formation of stacked packing layers, whereas the *ϕ*5 mm or *ϕ*8 mm balls form a single layer (Fig. 3a–c). For free balls in the packing layer, their velocity is slower than the confined ones within the layer (Fig. 3a, Movie S1). Thus, the free balls do not follow the rotation speed of the vessel. When the filling angle (*θ*) of balls from the vessel center exceeds 110–130°, the packing layers become stacked, and free-moving balls appear in the top layer. Upon loading 80 g *ϕ*8 mm balls, which is still below *θ* = 100°, a sliding motion is permitted in both the longitudinal and lateral directions perpendicular to the centrifugal force (Fig. 3f, Movie S2). A higher ball input results in a velocity distribution in the packing layer, decreasing from the wall to the center of the vessel (Fig. 3g–i). The collision between balls in a shearing direction (tangential direction) is dominant within the layers near the wall. Meanwhile, on the packing layers, the frequency of collisions in the normal direction increases due to an increase of free-moving balls. Thus, the collision direction and frequency change with the ball input in response to the packing state of the balls.

Next, we divide the total impact energy into the normal and tangential components. The tangential component has a higher impact energy per collision, while the normal one exhibits a wide distribution in the collision frequency (Fig. 2c). Most of the impact energy comes from the tangential direction, especially at a lower ball input (Fig. 2d). According to the predicted packing states, collisions in the tangential direction occur dominantly under the conditions where the sliding motion arises with a lower ball input. In contrast, the contribution of the normal component to the total impact energy increases from 7 to 36% with an increase in the input from 50 to 260 g for *ϕ*5 mm balls. The impact energy of the tangential component decreases when such collisions are constrained by surrounding balls in the stacked packing layers. With increasing ball input, since collisions in the normal direction increase due to the appearance of free-moving balls on the layer, the corresponding impact energy rises linearly. The collision frequency of the normal component is higher with smaller balls than with large ones (Fig. S2). The effect of collision frequency on the total impact energy appears at a very high ball input (Fig. 2d).

**Figure 4:**
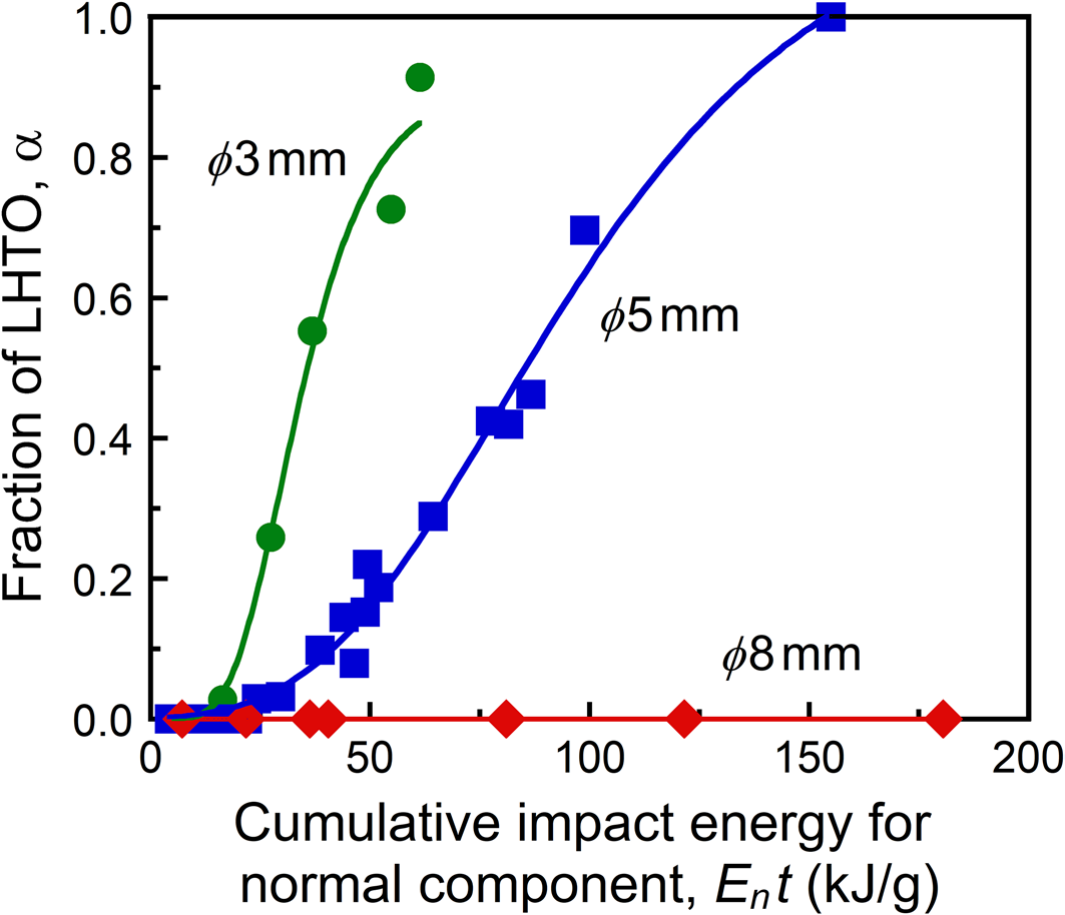
Effect of *E*_*n*_*t* on the formation fraction of LHTO through wet mechanochemical reaction.

Given the cumulative impact energies for each collision direction (*E*_*n*_*t* and *E*_*t*_*t* in normal and tangential components, respectively) based on the experimental conditions, key factors are revealed that influence the formation of LHTO in the wet mechanochemical reaction. We collected the formation ratios of LHTO obtained by varying the centrifugal acceleration, treatment time, and input for *ϕ*3 mm, *ϕ*5 mm, and *ϕ*8 mm balls because the minimum ball size that can be calculated by the developed simulation software is *ϕ*3 mm. Impact energies were calculated from simulations under the treatment conditions of the experimental data used. Furthermore, the impact energy under each condition was assumed to be per TiO_2_ input and the cumulative value of the treatment time. The fitting results demonstrate a good correlation between the formation fraction of LHTO and the cumulative impact energy of the normal component (*E*_*n*_*t*) at each ball size (Fig. 4), while there are no correlations with the impact energy of the tangential component (*E*_*t*_*t*) or the total impact energy (Fig. S3). Thus, we suggest that ball collisions in the normal direction strongly contribute to the formation of LHTO. The use of smaller balls promotes efficient formation of LHTO at lower *E*_*n*_*t* values. When fixing the conditions at *E*_*n*_*t* ≈ 60 kJ/g for *ϕ*3 mm and *ϕ*5 mm balls, the distribution of *e*_*n*_ per collision for *ϕ*5 mm balls nearly falls within that at *ϕ*3 mm (Fig. S4). Increasing the collision frequency in the normal direction promotes the formation of LHTO more efficiently. Unfortunately, LHTO formation could not be clearly observed when using *ϕ*8 mm balls, although a tiny hump in the XRD pattern is attributable to it at the condition of *E*_*n*_*t* ≈ 180 kJ/g (Fig. S5). From these results, we can conclude that increasing the collision frequency in the normal direction is more effective for product formation in the wet mechanochemical reaction, as compared to merely increasing the total impact energy of collisions. The reason why collisions of balls in the normal direction contribute to the wet mechanochemical reaction is not yet fully understood but is likely associated with different pressure-changes and volumes of solution/reactants caught between balls. The motion of balls in a shearing direction (tangential direction) generates a fluid flow, which seems to disperse the collision pressure and reactants. However, a more detailed analysis is needed in the future.

The data obtained with *ϕ*3 mm and *ϕ*5 mm balls follow the S-shaped reaction kinetics that is known as the Gompertz model^34^:

3 $$\upalpha = {\textit{A}\text{e}}^{{( - {\text{e}}^{{ - {\textit{k}}({\textit{E}}_{{\textit{n}}} {\textit{t}} -\uptau )}} )}}$$

where *A* is the asymptotic formation fraction, *k* is a reaction rate constant, and *τ* is the *E*_*n*_*t* origin. In general, such sigmoidal (S-shaped) dependence on the energy can be described by different models: the monomolecular, the autocatalytic, and the Gompertz models^34,35^. The curves of both the autocatalytic and the Gompertz models contain inflection points. Further, the autocatalytic and Gompertz curves are symmetric and asymmetric with regard to the inflection point, respectively. The Gompertz model provides a good fit to our data. An example of the Gompertz model is the growth of carbon nanotubes (CNTs)^36^. Carbon nuclei self-organized on a catalyst grow upward by thermal chemical vapor deposition and then form the CNTs^36–38^. A decelerating growth rate of CNTs could be due to catalyst deactivation, such as poisoning, overcoating, or evaporation. Similar to the growth model of CNTs, in the wet mechanochemical reaction, the formation of LHTO is driven by the impact energy of balls (especially in the normal direction), and the growth of LHTO driven by ion diffusion in the solution. The initial incubation/induction stage up to *E*_*n*_*t* ≈ 20 kJ/g is attributed to the step of TiO_2_ dissolution in LiOH solution. However, this step is difficult with *ϕ*8 mm balls. When the value of α is higher, its growth rate decreases due to certain bottlenecks such as a lower supersaturation, long-distance diffusion, or inhibition by impurities^39–41^.

Finally, although the rate of the wet mechanochemical reaction depends on collision in the normal direction, the impact energy is always higher in the tangential direction (Fig. 2c,d). The distribution of impact energy in the normal direction per collision mostly overlaps with that in the tangential direction (Fig. S2). However, there two energy components differ more at higher impact energies. Such excess impact energy should lead to wear in the balls and generate frictional heat. In comparison, mechanochemical reactions under dry conditions produce less impurity from the contact between balls because of the mediating or surface coating of powder reactants^42–44^. As explained in Eq. (1), an increased collision frequency of ball media in solution poses a problem from wearing. Possible ways to reduce impurities in wet mechanochemical reactions include: searching for milder milling conditions^16^, developing ball media that are more resistant to wearing^45,46^, or design the operating conditions or grinding mill to enhance collisions in the normal direction. When the collisions occur preferentially in the normal direction, one may achieve a high synthesis efficiency and suppression of impurities at the same time.

## Conclusion

In summary, the formation rate of LHTO through the wet mechanochemical reaction using a planetary ball mill is mainly controlled by collision of ball media in the normal direction. The collision of balls within the solution induces nucleation and crystal growth, and the use of smaller balls at high loading accelerates the product formation. Based on simulated ball motion during milling, we conclude that the formation of LHTO is not controlled by the tangential component, even though that component accounts for more of the total impact energy per collision. Rather, LHTO formation is mainly influenced by the normal component with a wide distribution of collision frequency. A remaining problem for the wet mechanochemical reaction is impurity generation due to the wear of balls, which may be due to the differential impact energy between the tangential and normal components. Future research on the milling conditions and/or the development of new grinding mills could improve the wet mechanochemical reactions by enhancing collisions in the normal direction, in order to effectively synthesize thermodynamically unstable materials at room temperature.

## Methodology

### Materials and synthesis of LHTO

LiOH·H_2_O (Kanto Chemical Co., Inc., Japan) and TiO_2_ (anatase, ST-01, Ishihara Sangyo Kaisha, Ltd., Japan) were used as starting materials. Their mixture (3 g, Li/Ti atomic ratio = 0.905) and pure water (30 mL) were placed in a stainless-steel vessel (170 cm^3^, inner diameter = 50 mm) containing Y_2_O_3_-stabilized ZrO_2_ balls (diameter, *ϕ*: 1–8 mm; input: 50–200 g; Nikkato Corp., Japan). The vessel was sealed and then processed in a planetary ball mill (High-G BX254E, Kurimoto Ltd., Japan). The milling was conducted at room temperature under centrifugal acceleration of 50, 100, and 150 G by controlling the revolution speed. The ratio of rotation/revolution speeds was fixed to 0.497. After milling for 1–10 h, the solid product was collected via centrifugation, washed several times with water, and dried in an oven at 100 °C.

### Characterization

Powder XRD patterns were collected on an X-ray diffractometer (D2 PHASER, Bruker AXS, Germany) using Cu *K*α radiation to identify the crystalline phase, with steps of 0.02° (2*θ*) and a counting time of 1 s/step. The fraction of LHTO formation was calculated from the intensity ratio between the strongest XRD peaks for LHTO and TiO_2_ (assuming no ZrO_2_) as follows:

4 $$ {\upalpha} = \left[ {I_{(200)}^{{{\text{LHTO}}}} /\left( {I_{(200)}^{{{\text{LHTO}}}} + I_{(101)}^{{{\text{TiO}}_{2} }} } \right)} \right]$$

### Simulation

We have developed a simulation software (KIK DEM, ver.1.1) to track the motion of ball media in a wet ball mill by DEM. The motion of spherical particle *i* (radius *r*) is determined by the force from the *k*_*i*_ particles it is in contact with. The equations of motion for translation and rotation are as follows:

5 $$m_{i} \frac{{{\text{d}}{\varvec{u}}_{i} }}{{{\text{d}}t}} = \mathop \sum \limits_{j = 1}^{{k_{i} }} \left( {{\varvec{F}}_{ij}^{n} + {\varvec{F}}_{ij}^{t} } \right) + {\varvec{F}}_{{\text{f}}} + {\varvec{F}}_{{\text{B}}} + m_{i} {\varvec{g}}$$

6 $$I_{i} \frac{{{\text{d}}{\varvec{\omega}}_{i} }}{{{\text{d}}t}} = \mathop \sum \limits_{j = 1}^{{k_{i} }} \left( {r_{i} {\varvec{F}}_{ij}^{t} } \right) + {\varvec{R}}_{{\text{r}}}$$

where *m* is the mass of a ball, *t* the time, ***u*** the translational velocity, *k* the number of particles, ***F***^*n*^ the normal force, ***F***^*t*^ the tangential force, ***F***_f_ the fluid resistance force, ***F***_B_ the buoyant force, ***g*** the gravitational acceleration, *I* the moment of inertia, ***ω*** the rotational velocity, and ***R***_r_ the rolling resistance. The parameters ${\varvec{F}}_{{{\textit{ij}}}}^{{\textit{n}}}$, ${\varvec{F}}_{{{\textit{ij}}}}^{{\textit{t}}}$, and ***R***_r_ are described in detail in ref. 24. The buoyant force was given by the volume of the ball media and the density of the fluid, while the fluid resistance force was given by the experimentally obtained equation acting on a single ball^25^. These interactions with fluids were considered through simplified modeling. When calculation the cumulative impact energies per unit mass under each experimental condition, we used only the mass of TiO_2_ because LiOH is soluble. Visualization of the simulated data was performed using ParaView software (ver. 5.8.0, Kitware Inc., USA, available: https://www.paraview.org).

## Supplementary Information

Supplementary Information 1

Supplementary Information 2

Supplementary Information 3

## Data Availability

The authors declare that relevant data are within the manuscript.
