# Supplementary material for: Effect of ball collision direction on a wet mechanochemical reaction
Source: Sci Rep. 2021 Jan 8;11:210. doi: 10.1038/s41598-020-80342-w (PMC7794608; doi:10.1038/s41598-020-80342-w)
Supplement: Supplementary file 1 — Supplementary Information 1 [file 41598_2020_80342_MOESM1_ESM.pdf]

Supporting Information

Effect of Ball Collision Direction on a Wet Mechanochemical Reaction

Takahiro Kozawa,[1,] Kayo Fukuyama,[1] Kizuku Kushimoto,[2] Shingo Ishihara,[2] Junya Kano,[2] Akira Kondo,[1] Makio Naito[1]*

1. Joining and Welding Research Institute, Osaka University, 11-1 Mihogaoka, Ibaraki, Osaka 567-0047, Japan

2. Institute of Multidisciplinary Research for Advanced Materials, Tohoku University, 2-1-1 Katahira, Aoba-ku, Sendai, Miyagi 980-8577, Japan

Corresponding Author

*E-mail: t-kozawa@jwri.osaka-u.ac.jp Tel: +81-6-6879-4366

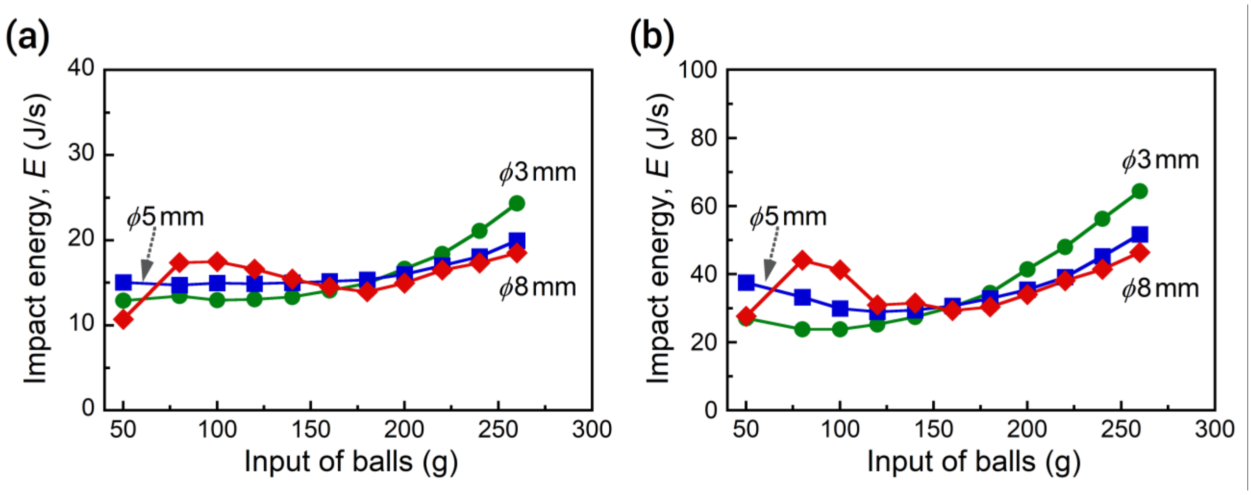

Figure S1. Relationship between the ball input (ϕ3–8 mm) and the total impact energies at (a) 50 G and (b) 100 G.

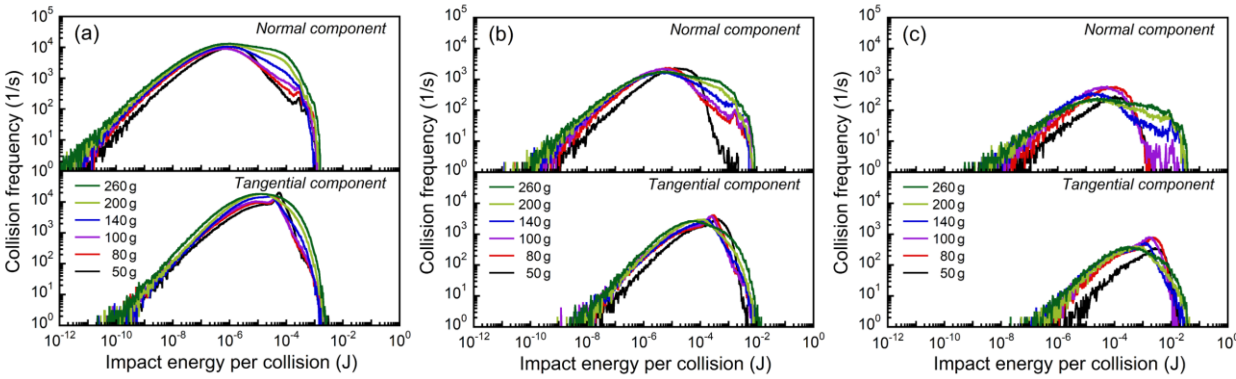

Figure S2. Distributions of impact energies in the normal and tangential directions at 150 G with (a) ϕ3 mm, (b) ϕ5 mm, and (c) ϕ8 mm balls and different inputs.

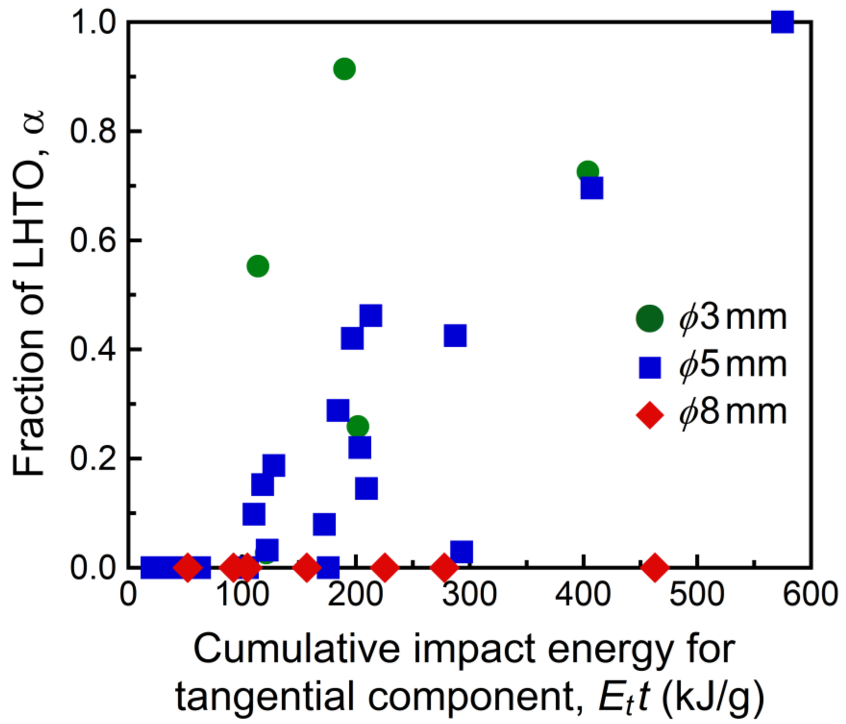

Figure S3. Relationship between $E_t t$ and the formation fraction of LHTO.

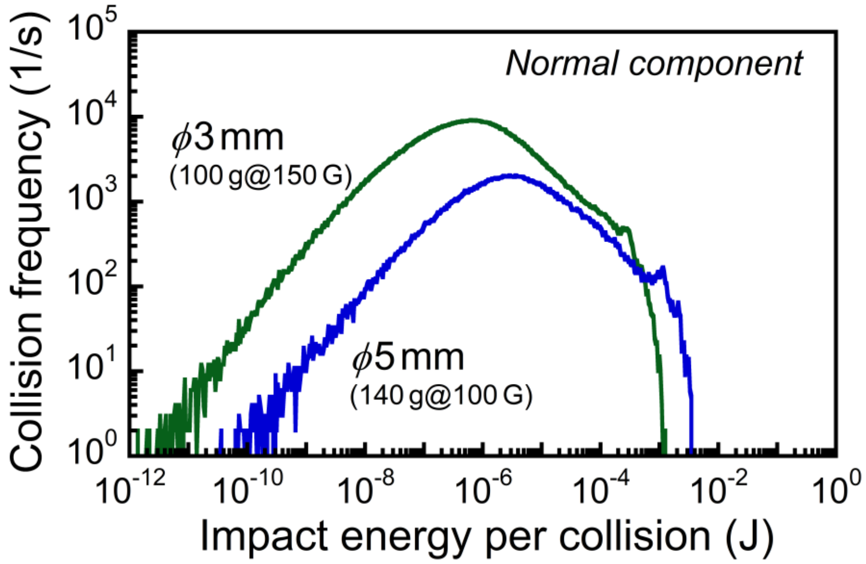

Figure S4. Distributions of impact energies in the normal direction under two milling conditions.

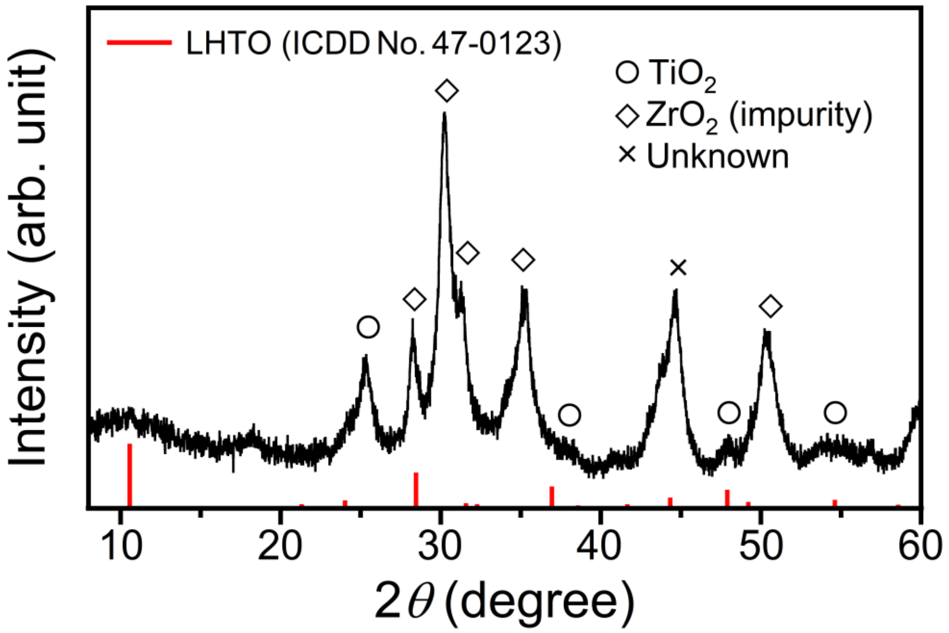

Figure S5. XRD pattern of the product obtained by milling at 150 G for 5 h with ϕ8 mm balls (180 g).
